# Hsp90 as a Gatekeeper of Tumor Angiogenesis: Clinical Promise and Potential Pitfalls

**DOI:** 10.1155/2010/412985

**Published:** 2010-06-24

**Authors:** J. E. Bohonowych, U. Gopal, J. S. Isaacs

**Affiliations:** Department of Cell & Molecular Pharmacology, Hollings Cancer Center, Medical University of South Carolina, 86 Jonathan Lucas Street, Charleston, SC 29425, USA

## Abstract

Tumor vascularization is an essential modulator of early tumor growth, progression, and therapeutic outcome. Although antiangiogenic treatments appear promising, intrinsic and acquired tumor resistance contributes to treatment failure. Clinical inhibition of the molecular chaperone heat shock protein 90 (Hsp90) provides an opportunity to target multiple aspects of this signaling resiliency, which may elicit more robust and enduring tumor repression relative to effects elicited by specifically targeted agents. This review highlights several primary effectors of angiogenesis modulated by Hsp90 and describes the clinical challenges posed by the redundant circuitry of these pathways. The four main topics addressed include (1) Hsp90-mediated regulation of HIF/VEGF signaling, (2) chaperone-dependent regulation of HIF-independent VEGF-mediated angiogenesis, (3) Hsp90-dependent targeting of key proangiogenic receptor tyrosine kinases and modulation of drug resistance, and (4) consideration of factors such as tumor microenvironment that pose several challenges for the clinical efficacy of anti-angiogenic therapy and Hsp90-targeted strategies.

## 1. Introduction

The concept of antiangiogenic targeting as a means to suppress malignancy came to the forefront of cancer therapeutics in the early 1970s, based upon the pioneering work of Folkman [1]. Tumor vascularization is a critical component of cancer progression, malignancy, and metastasis. As rapidly dividing cancer cells reach a critical tumor size, the mass outgrows its supply of blood, oxygen, and nutrients. Continued tumor growth depends upon the ability of microenvironmental stressors to trigger the activation of a complex and intricately interconnected signaling network that culminates in vascularization of the growing tumor. This activation process is essential for initiation of the “angiogenic switch,” a rate limiting event of tumor progression. Subsequent tumor vascularization culminates in either enhanced angiogenesis, the sprouting from local vessels, or vasculogenesis, the formation of neovessels through bone marrow-derived cell (BMDC) recruitment. Realization that the angiogenic switch may represent a universal Achilles heel for all tumors, coupled with elucidation of druggable targets within this network, has invigorated the field of antiangiogenic therapy, resulting in a rich pipeline of therapeutic compounds [2].

## 2. Emergence of Hsp90 Inhibitors as Antiangiogenic Chemotherapeutic Agents

Although a number of antiangiogenic therapies are presently in clinical use, the vast majority of these target a specific molecule or receptor family [3]. Despite a relatively high degree of specificity, the clinical efficacy of these therapies as curative agents remains poor. Though initial responsiveness may occur, the ultimate outcome is treatment resistance due to drug-dependent selection of intrinsic and adaptive resistance mechanisms. Therefore, attention has turned to chemotherapeutics targeting heat shock protein 90 (Hsp90), which simultaneously target multiple proangiogenic regulators, and may thus weaken the signaling resiliency characteristic of tumor cells. The prototypic Hsp90 inhibitor geldanamycin (GA) demonstrates potent antiangiogenic and antitumorigenic properties [4, 5]. At the molecular level, Hsp90 plays a critical role in the proper folding of its client or substrate proteins [6] and also serves as a scaffold protein to facilitate interactions between several receptor tyrosine kinases (RTKs) and their substrates. Therefore, Hsp90 inhibitors are unique as antiangiogenic agents in that they regulate the activity of hundreds of proteins, many of which support cancer growth [7–9]. In addition, Hsp90 expression is increased in many cancers, allowing sustained activation of cancer-specific dysregulated pathways and the buffering of stress conditions characteristic of the tumor microenvironment [10]. As a result, the evaluation of emergent Hsp90 inhibitors is a current focus of drug discovery efforts across multiple cancers [8, 9, 11].

The first discovered Hsp90 inhibitor, GA, is a naturally occurring benzoquinone ansamycin [12] that acts as a nucleotide mimetic to inhibit ATP-dependent Hsp90 chaperone activity. Although useful as a research tool, the pharmacological liabilities of GA prohibit its clinical use [13] leading to the development of subsequent generations of Hsp90 inhibitors including the GA analog 17-(allylamino)-17-demethoxygeldanamycin (17-AAG) and its water soluble version 17-dimethylaminoethylamino-17-demethoxy-geldanamycin (17-DMAG), both of which are being evaluated in multiple Phase I, II, and III clinical trials [14–16]. Alternatively, Hsp90 function and angiogenesis may be impaired by histone deacetylase (HDAC) inhibitors, that act upon the chaperone in a manner distinct from compounds that target its N-terminal ATP-binding pocket. Currently, the HDAC inhibitor LBH589/Panobinostat is under evaluation in Phase I and II trials [17]. The development of Hsp90 inhibitors, although in its infancy a decade ago, is now coming to the forefront of cancer therapy, with over 13 new entities being tested in a variety of preclinical models and human trials [18]. The antiangiogenic and antitumor effects of these agents will be discussed herein.

### 2.1. Rationale for Hsp90-Dependent Targeting of HIF

**Figure 1:**
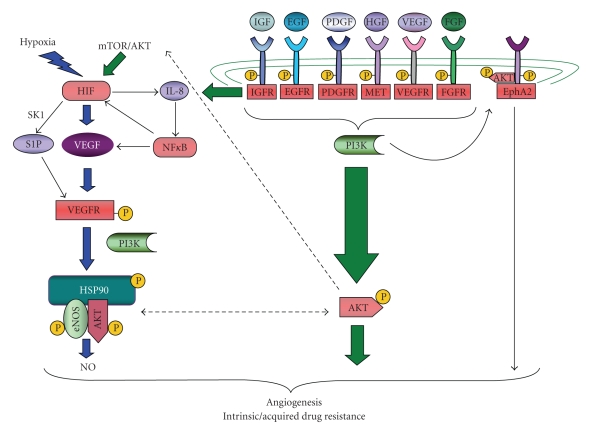
*Hsp90-dependent modulation of proangiogenic signaling pathways in cancer*. Hsp90 regulates multiple arms of angiogenic signaling in cancer. Key signaling molecules that are either direct clients or indirectly modulated by Hsp90 are shaded in red. One pathway that is commonly upregulated during tumorigenesis is the HIF/VEGF signaling axis. Tumor hypoxia and other stimuli induce HIF expression and subsequent activity, leading to a cascade of events that reinforce VEGF expression and angiogenesis. Importantly, several key mediators of this pathway, including HIF and VEGFR, are dependent upon Hsp90 for their function. As indicated, RTK activation also potently upregulates HIF via AKT/mTOR -mediated translation. RTKs additionally transactivate EphA2, a recently identified Hsp90 client protein known to participate in tumor vascularization. Providing another level of complexity, HIF also upregulates the expression of several RTK ligands (e.g., HGF and TGF-alpha), as well as RTK receptors (EGFR, IGFR), thereby reinforcing these signaling networks. Hsp90 additionally plays a role in NF*κ*B-dependent VEGF expression and regulates downstream effectors of VEGF signaling, including AKT-mediated eNOS phosphorylation. Given the intertwining levels among Hsp90 and angiogenic signaling cascades, Hsp90 intervention is predicted to impair signaling at many levels within these redundant pathways, with the overall effect of suppressing tumor angiogenesis.

Hypoxia inducible factor (HIF) is perhaps one of the most potent proangiogenic proteins regulated by Hsp90. Of the three known HIF isoforms, HIF-1 and HIF-2 contribute to cancer progression and their widespread overexpression in cancers correlates with increased mortality [19]. During tumor growth, HIF transactivates genes to favor survival under conditions of decreasing oxygen and nutrient availability [20]. A substantial number of these genes, such as vascular endothelial growth factor (VEGF) (Figure 1), fall within the category of proangiogenic cytokines, which collectively tip the scales in favor of angiogenesis and neovascularization as part of the angiogenic switch. Activation of this HIF-driven angiogenic switch releases growth constraints upon the tumor and sustains subsequent progression. Tumor cells have evolved multiple mechanisms for upregulating HIF expression and activity, most commonly via modulation of the HIF-*α* subunit, normally a tightly regulated labile protein subject to proteasomal degradation [21–23]. In general, these mechanisms block oxygen-dependent prolyl hydroxylase (PHD) enzymes from tagging HIF for degradation, ultimately preventing its proteasomal degradation via the von Hippel Lindau (VHL) ubiquitin ligase. VHL can also be inactivated via genetic mutation, genetic loss, or epigenetic suppression, all events most commonly associated with hereditary and sporadic clear cell renal cell carcinoma (CCRCC) [24, 25]. HIF overexpression correlates with highly vascularized tumors, resistance to chemo- and radiotherapy, and overall poor prognosis [26]. The essential role of HIF in tumor angiogenesis has been functionally validated in many models [27, 28], highlighting its importance and validity as a clinical target.

Although no specific anti-HIF targeting strategies have been approved, a number of currently utilized antiangiogenic agents have suppressive effects upon HIF activity or synthesis and diminish HIF-mediated VEGF expression [19]. Several of these agents exhibit antiangiogenic and antitumorigenic effects in preclinical models [29–32] and two specific HIF inhibitors are being evaluated in Phase I trials, the small molecule PX-478 [33], and the antisense oligonucleotide EZN-2968 [34]. The use of Hsp90 inhibitors as a strategy to target HIF emerged shortly after HIF was first identified as an Hsp90 client protein [35]. Many of the antivascular effects of Hsp90 inhibitors are likely due to the ability of this class of inhibitors to downregulate HIF activity. We and others have shown that both the HIF-1*α* and HIF-2*α* subunits are client proteins for Hsp90 and that chaperone activity is required for HIF stability and function [35–39]. Importantly, Hsp90 inhibitors, such as GA and its clinical derivative 17-AAG, promote proteasomal degradation HIF-*α* in a PHD/VHL and oxygen-independent manner, instead of utilizing the ubiquitin ligase RACK1 [37, 40–42]. Thus, Hsp90 inhibitors abrogate HIF signaling even in the absence of a functional PHD/VHL system, suggesting that these pharmacological agents may be able to dampen the constitutive HIF signaling associated with most solid tumors. This notion is supported by the ability of Hsp90 inhibitors to decrease VEGF secretion from cancer cells, impair endothelial cell tubule formation *in vitro*, and reduce *in vivo* tumor size and vascularization [43–45].

### 2.2. Hsp90 as an Effector of VEGF Expression and Angiogenic Activity

Although HIF is a main effector of VEGF-mediated signaling, HIF-independent proangiogenic processes also contribute to increased VEGF-dependent proangiogenic signaling [46]. NF*κ*B potently induces tumor vascularization, in part through its ability to upregulate VEGF expression through an IL-8/NF*κ*B signaling axis [47] (Figure 1). Secretion of IL-8, a potent proangiogenic factor, is mediated through a variety of mechanisms including other cytokines, cellular stress, and hypoxia (Figure 1) [48]. A positive feedback loop has also been reported whereby NF*κ*B can induce IL-8 and angiogenin secretion to promote tumor neovascularization through the recruitment of bone marrow derived cells [49]. Blockade of NF*κ*B signaling in an orthotopic model of ovarian cancer inhibited tumor growth, reduced tumor angiogenesis, and suppressed VEGF and IL-8 expression [50]. In addition to its role in HIF-independent upregulation of VEGF, NF*κ*B has also been shown to transcriptionally induce the physiological expression of HIF-1 [51]; however, it is not known whether this pathway may also exist in transformed cells. Although NF*κ*B itself is not a validated Hsp90 client protein, multiple direct modulators of NF*κ*B activation are subject to regulation by Hsp90, permitting Hsp90-inhibitor-mediated suppression of NF*κ*B [52–55]. Moreover, NF*κ*B may directly regulate Hsp90 expression through two binding sequences in the Hsp90 promoter, [56] suggesting the possibility of a feedback loop whereby Hsp90 inhibition decreases NF*κ*B activity, which may further decrease Hsp90 levels. It remains to be seen whether these HIF-independent angiogenic regulators of VEGF are appropriately suppressed upon Hsp90 inhibitor treatment in relevant preclinical models.

In addition to its involvement in both HIF and NF*κ*B mediated VEGF production, Hsp90 also modulates downstream effectors of VEGF-dependent signaling. VEGF mediates many of its proangiogenic effects through stimulation of the enzyme endothelial nitric oxide synthase (eNOS). In endothelial cells, activated eNOS leads to nitric oxide (NO) synthesis and release [57], which in turn promotes angiogenesis through endothelial cell proliferation and migration, as well as having effects on blood flow through modulation vascular tone and permeability [58, 59]. Mechanistically, VEGF binds to and activates the VEGF-R2 receptor, promoting phosphorylation of associated Hsp90 [60]. This phosphorylated Hsp90, in turn, serves as a scaffold to facilitate the association between eNOS and AKT, thereby promoting AKT-dependent phosphorylation and activation of eNOS (Figure 1) [61]. Microenvironmental factors such as hypoxia may facilitate angiogenesis by increasing the interaction between Hsp90 and eNOS concomitant with increased AKT activity in endothelial cells [62]. The association between eNOS and Hsp90 can be further stabilized by sphingosine-1-phosphate (S1P), a bioactive lipid induced during hypoxia in an HIF-dependent manner [63, 64], and reciprocally, S1P may stimulate HIF activity [65] and elicit chemoresistance [66]. Given that VEGFR, AKT, eNOS, and NF*κ*B share a dependence upon Hsp90, [67, 68] (Figure 1), Hsp90 inhibitors have the potential to target multiple steps of this pathway, as demonstrated by the potent suppression of VEGF and NO release both *in vitro* [69, 70] and in preclinical models [71, 72]. Additionally, 17-AAG directly inhibits transcription of eNOS mRNA in the *in vitro* angiogenic HUVEC (human umbilical vein endothelial cells) model, although the mechanism remains unknown [71]. Moreover, Hsp90 inhibitors decrease the expression of activated AKT and eNOS in HUVEC cells, concomitant with inhibitory effects upon tubule formation [72]. Therefore, the antiangiogenic properties of Hsp90 inhibitors are due in part to their ability to suppress HIF-dependent and independent VEGF expression and dampen the signaling potential of VEGF through modulation of VEGFR, AKT, and eNOS function. In light of these functions, Hsp90 chemotherapeutics have the potential to potently suppress tumor angiogenesis by attenuating the secretion of cancer-derived angiogenic factors as well as by blocking paracrine and autocrine signaling in the tumor-associated endothelium.

### 2.3. Hitting Hard: Hsp90 Inhibition as a Multifaceted Strategy to Combat Tumor Vascularity and Drug Resistance

Although VEGF plays a pivotal role in tumor angiogenesis and VEGF-targeted agents represent the cornerstone of many antiangiogenic approaches in malignant disease, the majority of these initially responsive tumors subsequently acquire drug resistance [3]. Broadly speaking, antiangiogenic tyrosine kinase inhibitors (TKIs) act by neutralizing VEGF/VEGFR signaling, VEGFR in combination with other proangiogenic RTKs, or target distinct RTKs that may not include VEGFR [3, 73, 74]. Common among all TKI-initiated strategies is a lack of durable response, an outcome that represents the biggest clinical challenge with TKI therapy. This universal property of therapeutic failure is a product of both *de novo* resistance, due to the inherent genetic complexity and heterogeneity of tumors, and to acquired resistance, a result of the plasticity and signaling redundancy characteristic of tumor cells [3, 75–77]. In the case of failure of VEGF-targeted therapy, the activation of compensatory mechanisms fosters adaptation and independence from VEGF signaling [78]. Compensatory and redundant signaling is a characteristic of several cancer types, such as glioblastoma multiforme (GBM), wherein activation of PDGF, MET, and EGFR family members collectively limits the therapeutic efficacy of specific TKI agents [79]. Importantly, the ability of angiogenic cytokines to activate their cognate RTK receptors is also a major determinant in conferring chemoresistance. Therefore, the salient finding that RTKs comprise the largest category of Hsp90 client proteins [7, 68, 80] (Figure 1) holds clinical promise for the ability of Hsp90-directed agents to suppress angiogenic signaling and overcome therapeutic resistance in diverse cancers. The following section will discuss Hsp90-modulated effectors of RTK driven angiogenesis, highlight several redundant signaling mechanisms contributing to drug resistance, and outline prospects for Hsp90 intervention and opportunities for reversal of this resistance.

The PDGF-FGF-VEGF signaling module represents a highly integrated pathway commonly activated in a number of cancers. The PDGF-*α* receptor participates in cell transformation, regulation of vascular permeability, and VEGF expression [81, 82]. Highlighting the complexity and interconnectedness of angiogenic signaling, PDGF also synergizes with FGF to stimulate neovascularization [83] and FGF, in turn, cooperates with VEGF to stimulate blood vessel maturation and function [84]. Clinically, several antiangiogenic TKIs are currently approved as inhibitors for these growth factor/RTK pairs, such as imatinib mesylate (gleevec), sorafenib, and others [85]. The interconnectedness of these pathways suggests a potential benefit of combining TKIs in antiangiogenic therapy, supported by the clinical observation that combination of a pan-VEGFR inhibitor (sunitinib) with a PDGF inhibitor (AGO13736) delayed tumor progression more effectively in cytokine refractory renal cancer [86]. The EGF-IGF-HGF-VEGF signaling module represents another proangiogenic molecular hub. Activation of the MET RTK receptor by its ligand, hepatocyte growth factor (HGF), which may be upregulated by a HIF-dependent pathway [87], has pleiotropic angiogenic effects including upregulation of VEGF and its receptor [88], as well as stimulation of HIF-mediated VEGF secretion [89]. MET also cooperates with epidermal growth factor receptor (EGFR) through the downstream effectors PI3K/AKT [77]. EGFR family members EGFR and HER2 are overexpressed in many cancers and are integral to tumor progression, in part due to their ability to stimulate release of angiogenic factors including VEGF [90]. Continuing the theme of cross-communication, EGFR and HER2 cooperate with IGFR through the PI3K pathway to synergistically increase vessel growth [91]. Although cancer cells harboring amplified MET are initially sensitive to MET TKIs *in vitro*, they evade this inhibition, despite durable MET inhibition, via reactivation of EGFR and downstream mediators [92]. Antiangiogenic TKIs targeting these receptors are being evaluated in Phase I and II clinical trials [3].

The targeting of EGFR with gefitinib provides a well-characterized scenario illustrating the pleiotropic mechanisms at play in drug evasion. One common mechanism for therapeutic escape is conferred by secondary mutations in RTKs that impair the ability of targeted therapeutics to recognize and block receptor function. For example, although at least three EGFR TKIs are in clinical trials (imatinib, gefitinib, and erlotinib), resistance occurs with a frequency of 70% in lung cancer patients, due to the acquisition of secondary mutations in the kinase domain [76, 93]. Another caveat is that gefitinib treatment results in the activation of signaling pathways that collectively serve to limit its antitumor activity and allow acquired resistance to emerge. A number of these resistance mechanisms have been identified in both preclinical and clinical studies and include activation of oncogenic signaling, HGF, VEGF, and PI3K/AKT activation, the latter of which plays a role in *de novo* resistance and sustenance of EGFR activation in a number of cancers [77, 85, 94–98]. A number of studies implicate the contribution of IGF-1R and FGFR as participants in both intrinsic and acquired resistance to EGFR TKIs [98]. These findings strongly advocate the use of combination therapy as a means to overcome this signaling redundancy and subsequent drug resistance. In support of this notion, it was demonstrated that coadministration of gefitinib and an IGF-1R inhibitor (NVP-AEW541) in a gefitinib resistant xenograft model reversed drug resistance [99]. Other reports also document the importance of cotargeting downstream molecules, including the Hsp90 regulated effectors PI3K/mTOR and MEK, in regaining drug responsiveness in EGFR mutant cancers [98]. These examples highlight the large scope of compensatory mechanisms utilized by tumor cells and the need for broad acting agents and/or combinatorial approaches such as those represented by Hsp90-directed chemotherapeutics.

The inherent ability of Hsp90-targeted agents to suppress multiple proangiogenic receptors and effector proteins bodes well for their prospects in overcoming the redundancy of signaling required for adaptive responses. In support of this premise, geldanamycins (GAs) destabilize MET, inhibit its angiogenic function [100], and suppress coordinate signaling effectors that elude receptor-specific targeting strategies [92]. Blocking Hsp90 also inhibits IGFR and PDGFR function and reduces their angiogenic potential *in vivo* [45, 101]. Similarly, GAs suppress EGFR/HER2 signaling, impair multiple pathways associated with receptor upregulation, and reduce tumor growth and vascularization *in vivo* [45, 102, 103]. Importantly, TKI resistant EGFR mutant receptors are exquisitely sensitive to Hsp90 inhibitors and these agents synergize with EGFR inhibitors [104]. Interestingly, clinical administration of 17-AAG led to tumor regression in HER-2 positive metastatic breast cancer patients [105], demonstrating the oncogene-addicted nature of this tumor. The basis for the poor response in HER-2 negative subtypes remains unknown, given that Hsp90 inhibition also cotargets pivotal downstream effectors of RTK signaling, such as FAK, MAPK, and AKT [67, 106, 107], which would be expected to amplify their suppressive effects and thwart adaptive responses. This ability of Hsp90 inhibition to fundamentally alter network circuitry in tumor cells bodes well for their ability to potentiate the efficacy of targeted or cytotoxic drugs administered in tandem, a notion supported by its synergistic antitumor and antivascular in numerous *in vitro* and preclinical models [104, 108–112].

## 3. EphA2 Receptor as a Conduit for RTK Activation and a Driver of Angiogenesis

The Eph RTK superfamily is of recent interest in relation to proangiogenic proteins targeted by Hsp90-directed therapy. Several of the 16 Eph RTKs demonstrate functions pertaining to vessel development during embryogenesis and in cancer [113]. In particular, the EphA2 receptor is emerging as a pivotal regulator of physiological and pathological angiogenesis. EphA2 plays an essential role in developmental angiogenesis, as null endothelial cells fail to undergo cell migration and vascular assembly both *in vitro* and *in vivo* [114]. A definitive role for EphA2 in malignancy is illustrated by diminished tumor growth, angiogenesis, and metastasis in EphA2 deficient mice [115]. Furthermore, EphA2 is commonly detected in the tumor associated vasculature, where it facilitates angiogenesis [116–118]. EphA2 is overexpressed in a number of human malignancies, particularly in highly vascular GBM tumors, where it serves as a prognostic factor [119]. Unlike most other ligand/RTK interactions, the association of ephrinA1 ligand with the EphA2 receptor is inhibitory, due to subsequent internalization and proteasomal degradation of the receptor [113, 120]. In cancers, ligand expression is downregulated during the malignant process [119], leading to constitutive EphA2 signaling. In terms of therapeutic options, no clinical TKIs against EphA2 have yet been developed, nor have any already approved clinical agents been shown to target EphA2. However, targeting of EphA2 signaling by either siRNA-mediated suppression of receptor expression [121] or administration of a selective antibody [122] are approaches that have demonstrated efficacy in preclinical models. Highlighting an alternative therapeutic approach, we found that EphA2 receptor activity requires Hsp90 function [123] and, further, that Hsp90-targeting agents interfere with EphA2-mediated signaling and promote receptor destabilization. Therefore, Hsp90 inhibition may represent a therapeutic approach to neutralize receptor function and reduce the aggressiveness of EphA2-driven cancers.

In keeping with the theme of cross-communication, EphA2 is earning its place as an essential member of an expanding network of protumorigenic effectors. First, EGFR activation drives MAPK signaling, which leads to its upregulation in a number of aggressive cancers [124]. Secondly, EGFR family members co-opt EphA2 to promote cell motility and proliferation [115, 125]. Third, EphA2 also cooperates with VEGFR, as demonstrated by its requirement for VEGF-induced endothelial cell migration and tubule formation [126]. Finally, EphA2 function is transduced by its ability to complex with AKT, the latter of which is activated by diverse RTK ligands (EGF, FGF, HGF, and PDGF) [120]. This multifaceted mechanism for EphA2 activation reinforces the theme of the previous section in that the effective blockade of EphA2 signaling will require the simultaneous cotargeting of multiple RTKs. Growth factor-mediated AKT phosphorylation in turn leads to AKT-dependent EphA2 phosphorylation, which facilitates EphA2/AKT complex formation (Figure 1). Formation of this signaling unit is critical for tumor cell migration and invasiveness. Although it remains unclear how this pathway mechanistically translates to angiogenic potential, AKT is a known mediator of angiogenic processes [127] and AKT-mediated EphA2 phosphorylation within GBM-associated tumor vasculature increases with malignancy [120]. Given that AKT is a validated Hsp90 target protein [128] activated in a number of malignancies, and that AKT acts as a conduit for EphA2 signaling, Hsp90-targeting strategies should be effective in blocking EphA2/AKT-dependent angiogenesis. Interestingly, most of the aforementioned angiogenic cytokines are also reported to stimulate VEGF secretion, due in part to their ability to stimulate HIF translation via mTOR, a downstream effector for AKT. As recently reviewed [19], many of the aforementioned TKIs also downregulate HIF translation. Collectively, cytokine-mediated activation of RTKs, and subsequently of AKT, stimulates HIF translation, thereby potentiating VEGF-dependent signaling and sustained angiogenesis (Figure 1). In a recent twist, it was shown that HIF-2 regulates both the expression and activation of multiple RTKs [129]. Furthermore, the suppression of HIF dramatically improves tumor responses to Sunitinib in colon cancer cells and strikingly prolonged complete responses in half of the tumor bearing mice [130]. This demonstrated ability of HIF to regulate RTK signaling and potentiate the effects of antivascular agents further emphasizes the complexity and cross-pollination of these signaling pathways and supports the rationale for utilizing Hsp90-targeted agents as a strategy to cotarget HIF proteins and proangiogenic RTKs.

## 4. Murky Waters: Clinical Challenges of Hsp90 Inhibition

Although Hsp90 sustains a multitude of angiogenic processes critical for cancer progression, and Hsp90-targeted agents demonstrate favorable responses across multiple cancers in preclinical models, they have fared less well in the clinic. Recent clinical failures include the use of 17-AAG in advanced prostate cancer and CCRCC [131, 132], the latter a particularly surprising outcome given the putative HIF-dependent and angiogenic nature of this tumor. However, these patients had already presented with advanced metastatic disease, and it is therefore unclear whether improved responses might have been observed with earlier intervention. This section will evaluate some of the more complex issues and potential pitfalls that may offer insight into the variable clinical response of these inhibitors, including pharmacologic considerations, undesired effects of Hsp90-directed therapy, and role of the tumor microenvironment.

### 4.1. Toxicity, Metabolism, and Delivery

Discordance between Hsp90-targeted efficacy in preclinical models compared with less favorable clinical outcomes may be due to a number of pharmacologic factors, independent of the ability of these agents to target the appropriate protumorigenic pathways. One issue may pertain to drug formulation, as 17-AAG/Tanespimycin is a substrate for the multidrug resistance (MDR) transporter P-glycoprotein, and the related MRP efflux pump [133]. Acquired resistance of cells via this mechanism has been observed in cell culture [134]. Another caveat is that drug potency requires reduction by NAD(P)H:Quinone Oxidoreductase I (NQO1) [133, 135, 136]; yet information is lacking on NQO1 expression profiles in treated patients. A new generation of purine-based Hsp90 inhibitors was subsequently developed [137] whose activity does not depend upon this reduction event [134]. Purine-based compounds are currently at the forefront of Hsp90 inhibitor advancement with several derivatives exhibiting increased potency and decreased toxicity when compared to 17-AAG [138]. Furthermore, these agents are not subject to metabolism by NQO1/DT-diaphorase enzymes nor to efflux by P-glycoprotein, and tumor cells in culture that have acquired resistance to 17-AAG remain susceptible to these newer agents [134, 139]. Therefore, despite disappointing early clinical results with 17-AAG, clinical enthusiasm for the next generation of Hsp90 inhibitors remains high.

### 4.2. Molecular Caveats of Hsp90 Inhibition

In addition to drug formulation challenges, inhibition of cellular Hsp90 initiates a heat shock response [140] that triggers activation of heat shock factor 1 (HSF-1) [140] and corresponding upregulation of prosurvival chaperones Hsp27, Hsp70, and Hsp90. This heat shock response antagonizes drug potency *in vitro* and *in vivo* [140–143]. Furthermore, HSF-1 expression is essential in supporting malignant transformation [144]. A heat shock response is similarly initiated with the newer purine derivatives [139, 142] and therefore represents a characteristic associated with this general class of Hsp90 targeting agents. However, it may be possible to address this undesired effect through combination treatments. For example, cisplatin suppresses 17-AAG-mediated HSF-1 activation and synergistically promotes tumor cell death *in vitro* [145]. It remains to be seen whether clinical modulation of the HSF-1-mediated heat shock response may enhance the clinical efficacy of Hsp90-directed therapy.

Another less well-understood consideration involves molecular factors that may alter the efficiency of Hsp90-mediated client destabilization. Many Hsp90 client proteins are destabilized via the concerted action of ubiquitin ligases and the proteasomal pathway; however little is known about the molecular effectors involved in client destabilization following Hsp90 inhibition. Taking HIF as an example, we have previously demonstrated that the association of HIF with its dimerization partner ARNT promotes dissociation of Hsp90 from HIF, with resultant protection from Hsp90 inhibiting agents [40]. Since that report, others have documented additional HIF binding or accessory proteins that may share a similar propensity to modulate the efficiency of Hsp90 directed HIF degradation [42, 146, 147], highlighting the previously unappreciated molecular complexity associated with Hsp90-targeted therapy. Recently, a group demonstrated that expression of Cullin5, a ubiquitin ligase, was required for optimal degradation of HIF and ErbB2 [148]. Importantly, Cullin5 expression is decreased in an overwhelming majority of breast cancers [149], suggesting that tumor cells may acquire the ability to evade or limit the destabilization effects of Hsp90-dependent chemotherapy upon subsets of clients. The identification of these modifiers and an analysis of their expression in human cancers may be required to gauge the clinical potential of Hsp90-directed therapy against specific clients or signaling pathways.

### 4.3. Fighting a Tiger: Contribution of Stroma to Tumor Angiogenesis and Malignant Progression

**Figure 2:**
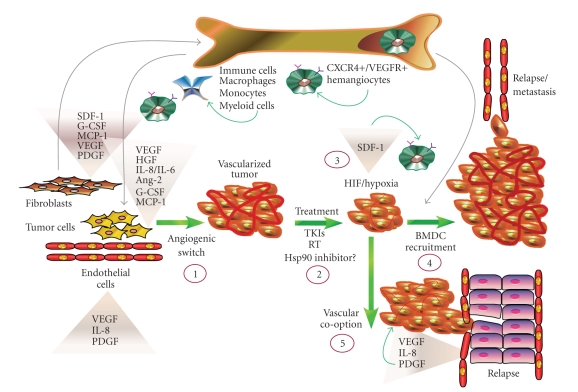
*Potential for Hsp90 intervention strategies in curtailing angiogenic processes*. Tumor stromal cells, such as cancer-associated fibroblasts (CAFs) and endothelial cells (ECs), communicate with tumor cells via their secretion of cytokines and thus contribute to the angiogenic switch (1). Cytokines from recruited BMDC progenitors contribute to this milieu to further stimulate tumor vascularization (1). Hsp90 inhibition may prevent HIF-driven cytokine release from tumor and stromal cells (i.e., SDF-1, VEGF, HGF, etc.), as well as HIF-mediated CXCR4+ expression in BMDCs, with the potential effect of curtailing recruitment of CXCR4+ progenitors to the tumor. Hsp90 inhibition also attenuates cytokine signaling via RTK inhibition (i.e., VEGFR, PDGFR), which may collectively prevent or delay the angiogenic switch. Therapeutic approaches utilizing radiotherapy (RT), tyrosine kinase inhibitors (TKIs), or Hsp90-targeted agents suppress tumor vascularization and growth (2). This initial reduction in vascularity may promote tumor hypoxia (3), subsequent HIF activation, and SDF-1 secretion, the latter of which may further stimulate BMDC recruitment (4). Hsp90 inhibitors are similarly predicted to suppress BMDC recruitment and HIF-driven cytokine secretion as in (1). Alternatively, when challenged with reduced HIF expression and decreased BMDC recruitment, tumor cells may coopt the vasculature of normal tissue (5). In this scenario, Hsp90 suppression is predicted to reduce the efficiency of EC-derived factors that support this process. The overall efficacy of Hsp90 inhibition upon tumor vascularization will depend upon the balance of Hsp90-dependent and Hsp90-independent signaling effectors driving the angiogenic process.

Another factor in the disconnect between preclinical results and efficacy in patients is likely attributed to the influence of the tumor-adjacent stromal tissue. The tumor stroma, which influences the microenvironment surrounding the neoplastic lesion, is a driving force of both tumor growth and vascularization. This heterogenous stroma is comprised of a variety of cell types, including fibroblasts/myofibroblasts, smooth muscle cells and endothelial cells, immune cells, and bone marrow progenitor cells (Figure 2) [150], all of which collaborate to spur tumor growth. Clinically, an abundant fraction of the tumor reactive stroma is composed of cancer-associated fibroblasts (CAFs), or myofibroblasts, whose presence is recognized as a risk factor for neoplastic transformation. This tumor supporting function is due to their ability to upregulate ECM proteins and secrete growth factors and cytokines, (i.e., fibroblast growth factor (FGF), connective tissue growth factor (CTGF), stromal-derived factor (SDF-1) and VEGF), thereby creating conditions that constitute an optimal milieu for tumor development [151, 152]. CAFs robustly stimulate tumor angiogenesis and cancer progression in a number of xenograft models [153–157]. Myofibroblasts are also a component of nontumorigenic fibrotic tissue, such as in gastric, pancreatic, and hepatic stellate cells, which similarly secrete angiogenic cytokines and support paracrine signaling in adjacent epithelial cells [158]. Hypoxic signaling further drives fibrogenesis [159], and the hypoxia-mediated upregulation of VEGF and angiogenic cytokines from stellate cells perpetuates the fibrotic and hypoxic cycle [158, 160].

Given that myofibrolasts support and sustain cancer progression, there is heightened interest in agents that may target the CAF-rich stroma [161]. Although Hsp90-targeted strategies are predicted to interfere with the VEGFR, PDGFR, and TGF beta receptor signaling that participates in tumor-stromal communication, reports examining the ability of either TKIs or Hsp90 inhibitors to attenuate CAF/stellate signaling are limited. Multi-TKIs reverse properties associated with the activated stellate phenotype *in vitro* and *in vivo* [162, 163], concomitant with reduced tumor vascularity [164], and Imatinib inhibits PDGF/AKT signaling and ECM production in breast stromal fibroblasts [165]. Hsp90 inhibitors exhibit cytotoxicity against CAFs derived from gastric cancer [44], which are potent mediators of tumor angiogenesis [166]. Interestingly, Hsp90 inhibition suppresses the reactive stromal phenotype in hepatic stellate cells, prior to initiating apoptosis [167], suggesting that Hsp90-targeted agents may be effective in reducing stromal support of tumorigenic progression.

Given that CAFs promote tumor vascularization in part via their ability to stimulate endothelial cell function, attention has now shifted to the ability of TKIs to cotarget the tumor endothelium. The stromal vasculature is comprised of endothelial cells and perivascular pericytes, the latter of which provide both survival signals and structural support to facilitate a mature and functional vasculature [168]. The pathways crucial to EC-pericyte communication and vascular stabilization include PDGF/PDGFR and VEGF/VEGFR signaling. Secretion of PDGF from cancer cells stimulates VEGF upregulation in pericytes via a PI3K/AKT mechanism, which protects ECs from apoptosis [169]. Furthermore, PDGF signaling is essential for tumor vascularization in a preclinical model of pancreatic cancer [170] and upregulated PDGF signaling is a characteristic of the tumor stroma in a preclinical model of cervical cancer [171]. In the latter example, expression of the PDGF receptor was primarily localized to stromal fibroblasts and pericytes, and inhibition of PDGFR signaling with Imatinib decreased pericyte coverage of tumor endothelial cells, concomitant with suppression of tumor vascularity and growth.

Hsp90 inhibitors are well characterized in their ability to suppress tumor vasculature and growth in multiple preclinical models; yet their specific effects upon endothelial cells and pericytes remain largely unexplored. A limited number of reports demonstrate that Hsp90 inhibition reduces proliferation, differentiation, motility, and angiogenic signaling in normal human endothelial cells [72, 139] and may elicit death of normal vascular smooth muscle cells (representative of pericytes) at high concentrations [44], but the molecular mechanisms for these effects remain obscure. The precise molecular effects of Hsp90 inhibition within the tumor-associated vasculature have not been clarified, and use of normal cells that resemble components of the tumor vasculature may not accurately reflect the tumor endothelium. Further, Hsp90 inhibitors may differentially destabilize proteins in tumor and vascular cells [101], illustrating the potential for differential tumor and stromal responses to therapeutics. Importantly, Hsp90 therapeutics may exhibit preferential uptake and selectivity for tumor tissue, proportional to the cancer specific hyperactivity of the Hsp90 chaperone [172, 173], inviting the question of whether these agents will effectively target components of the nontumorigenic stroma.

### 4.4. The Tumor Stroma as an Enabler of Therapeutic Resistance

Clinically, chemoresistance may be due to a failure to target the proangiogenic signaling derived from tumor stroma, a notion supported by the ability of CAFs to mediate drug sensitivity [174, 175]. Endothelial cells also contribute to chemoresistance, as demonstrated by the use of temozolomide in the treatment glioblastoma multiforme (GBM). While temozolomide exhibits toxicity against GBM cells in culture, glioma-derived endothelial cells are refractory to treatment, thus offering a possible explanation for the clinical failure of these agents [176]. Similarly, the ability of pericytes to protect against EC apoptosis [169] implicates their potential role in attenuating the efficacy of chemotherapeutics. In support of this notion, dual inhibition of PDGFR and VEGFR signaling is synergistic in reducing pericyte coverage of tumor endothelium, and this combination is maximally effective in suppressing tumor growth over either agent alone in a mouse model of pancreatic islet cancer [177]. In addition to its role in chemoresistance, the tumor stroma also plays a major role in mediating the response to radiotherapy (RT). It is established that RT induces the expression and secretion of VEGF and proangiogenic cytokines in cancer cells, stromal endothelial cells, and fibroblasts [178–180]. This cytokine upregulation stimulated by the initiation of vascular repair mechanisms and HIF-dependent signaling [181, 182] represents a major barrier to chemosensitivity [183–185].

Antiangiogenic TKIs have met with limited success in combating radioresistance, despite their impairment of proangiogenic RTK signaling in both tumor and stromal cells [186]. Although not yet validated clinically, we, and others, have shown that Hsp90 inhibition imparts radiosensitivity to various cancer cell types in culture and *in vivo* [187–189]. While the basis for this enhancement is not well defined, Hsp90-dependent HIF targeting is one likely mechanism, supported by the finding that blockade of HIF/VEGF signaling, coupled with RT, potently destroys tumor vasculature in a xenograft model [190]. In further support of the ability of Hsp90 inhibition to impair this signaling axis, Hsp90-directed therapy overrides the protective effects of VEGF-mediated signaling in endothelial cells, in part through inhibition of AKT [191]. Since many of the RT-induced wound repair response pathways depend upon a number of Hsp90 clients, including HIF, AKT, MEK/ERK, PDGFR, and VEGFR, Hsp90 inhibition represents an alternate approach to impair multiple radioresponse regulatory proteins and improve outcome. In fact, it remains a possible, though unconfirmed notion, that the ability of Hsp90 inhibitors to destabilize interactions between ECs and pericytes may underlie their ability to suppress tumor vascularity and potentiate chemo- and radiosensitivity. However, given that optimal responses to the combination of anti-VEGF therapy with RT occur during the “normalization window” characterized by stabilization of the tumor vasculature subsequent to enhanced pericyte coverage [192], the use of Hsp90 inhibitors will have to be judiciously applied for optimal therapeutic benefit.

Similar to RT, vascular targeting agents exacerbate tumor hypoxia and activate HIF signaling, events antagonistic to treatment objectives. HIF activation stimulates cytokine secretion and recruitment of circulating BMDCs (Figure 2), which may replenish vascular components such as pericytes, CAFs, and myeloid cells. Mobilization of BMDCs to hypoxic areas promotes neovascularization and contributes to cancer aggressiveness and drug resistance [2, 193, 194]. Although factors regulating BMDC recruitment are not well defined, tumor hypoxia and the HIF-regulated cytokines VEGF and SDF-1 are established as major effectors in the recruitment of VEGFR and CXCR4 expressing BMDCs [49, 195, 196] (Figure 2). In a well-validated preclinical model, hypoxic or irradiated glioma cells each secreted factors that stimulated the homing of hematopoietic progenitors in a HIF-dependent manner [197]. The enhancement of tumor hypoxia via genetic or pharmacologic interference with VEGF signaling similarly recruited BMDCs and fueled aggressiveness [198, 199] (Figure 2). It remains to be seen whether Hsp90-directed therapy may effectively suppress HIF-mediated recruitment of BMDCs and whether attenuated recruitment may be sufficient for tumor suppression. Indeed, BMDC recruitment was diminished in a HIF-1 knockout mouse model of glioma; yet tumor cells became invasive and continued to thrive via cooption of the host vasculature [200]. In this example, the host cells were still competent to respond to tumor-mediated HIF/VEGF signaling, leaving open the possibility that Hsp90 inhibition, which would target HIF signaling in conjunction with VEGF mediated responses, may thwart this undesired adaptive event (Figure 2).

Another level of complexity is due to the nature of angiogenic compensation, in that HIF-independent compensatory mechanisms, such as NF*κ*B-dependent cytokine release, can also promote BMDC recruitment [49]. Hsp90 inhibitors have the potential to impair a subset of HIF-independent mechanisms, for example, by virtue of their ability to suppress effectors such as NF*κ*B. Although the role of Hsp90-dependent signaling within the context of BMDC-mediated neovascularization has not been explored, the ability of Hsp90 inhibitors to suppress BMDC stimulatory properties upon multiple myeloma cells in a coculture model [72] suggests that these agents may indeed have utility in preventing some aspects of BMDC-derived neovascularization. However, these agents are likely to incur a number limitations, given the recent identification of a subset of myeloid cells that are refractory to VEGF-dependent therapy, due to expression of G-CSF and Bv8, the latter of which drives local angiogenesis, as well as BMDC recruitment [199, 201]. As these ligands activate G-coupled protein receptors (GPCRs) that may not rely upon Hsp90 function, it is possible that this alternative angiogenic pathway will be immune to Hsp90-directed therapy. It therefore remains to be determined whether the lack of durable clinical response of Hsp90-directed therapy is due to an inability to suppress BMDC recruitment, to inhibit vascular cooption, or to suppress these compensatory angiogenic pathways. A further characterization of these pathways may pave the way to more successful regimens that employ rationally designed combination therapies.

### 4.5. The Road to Nirvana: Improving Preclinical Models, Drug Analyses, and Clinical Correlates

With the overarching goal of understanding the basis for clinical responses to Hsp90-targeted therapy, a continuum of models must be evaluated, each with its inherent strengths and weaknesses. Although cell-based *in vitro* models are necessary to decipher defined molecular signaling events, these systems do not recapitulate the complexity of the tumor microenvironment, such as dimensionality, matrix incorporation, or stromal communication, all factors that influence antiangiogenic responses [202, 203]. Xenograft models, which embody much of the complexity of the tumor microenvironment, are a necessary prerequisite for evaluating the utility of antiangiogenic agents in distinct cancers. However, the majority of these models utilize immunocompromised mice, which lack the full complement of immune components that may contribute to stromal dependent angiogenesis. Some of these differences invariably account for the ability of Hsp90 inhibitors, and other chemotherapeutics, to exhibit more potent tumor efficacy in preclinical models compared with their clinical responses. Immune competent genetic models of progressive cancer that more closely mimic the complexity of human malignancy represent valuable tools for dissection of stepwise genetic events in this process and may also help pinpoint when antiangiogenic agents would be most efficacious.

Another caveat to deciphering optimal treatment regimens is imparted by the molecular heterogeneity of cancers. Functionally, this is exemplified by the differential wiring of signaling networks, and the corresponding differential response to Hsp90 inhibition, a trait similarly reflected with other chemotherapeutics. These varying responses likely reflect the distinct molecular signatures driving cancer survival, each with a unique dependence upon Hsp90 function [204], combined with disparate local microenvironments from the periphery to the core. Moreover, differential client affinity in diverse cell and cancer types [205] hints at more complex molecular regulation. Further, the recent revelation that an average human tumor may have upwards of 20,000 mutations [206, 207] strongly supports the rationale for therapeutic strategies incorporating more broadly acting drugs, such as those represented by the category of Hsp90-directed agents. In addition to tumor cell heterogeneity, components of the tumor vasculature may demonstrate comparable complexity. In support of this notion, VEGF resistant CAFs may upregulate PDGF signaling [175] and promote chemoresistance. Similarly, the tumor endothelium exhibits significant heterogeneity of expressed surface molecules, as validated with use of phage display technology, based upon the principle of differential homing of peptides to the vasculature [208–210]. Interestingly, this approach identified distinct molecular signatures of the tumor endothelium that occur in a stage specific manner, with clinical implications for the variable tumor responses observed for TKIs. In a preclinical model of cervical cancer, a subset of TKI agents demonstrated preferential activity at distinct tumor stages, implicating their selective effects upon either tumor initiation or signaling events activated at later stages [171]. In this model, combination treatments (i.e., cotargeting VEGFR and PDGFR) demonstrated improved antitumor activity of progressive tumors, illuminating that optimal targeting approaches may need to consider prevention and intervention strategies as a component of the heterogeneity of the tumor-associated vasculature. Similar dynamic treatment analyses in well-defined preclinical models will further define optimal clinical usage for Hsp90 inhibitors.

In addition to allowing a dynamic evaluation of drug efficacy, preclinical models are useful tools to assess complex drug-dependent molecular changes. A subset of microRNAs was recently identified as components of the angiogenic switch in a genetic model of pancreatic cancer. Strikingly, these same targets were similarly upregulated in patients and modulated preclinically following treatment with Sunitinib [211]. Although a comparative detailed genetic analysis has not yet been performed with Hsp90 inhibitors, one group evaluated pharmacodynamic markers and resultant tumor angiogenesis [139], while another performed a proteomic and genetic analysis to identify a number of gene and protein changes [212]. Further preclinical models that incorporate dynamic molecular analyses have great potential for evaluating the effects of emerging Hsp90 inhibitors. Similarly, clinical trial design is likely to benefit from the incorporation of increasingly sophisticated molecular endpoints. Although the evaluation of changes in tumor burden and disease progression are established components of the clinical evaluation of anticancer agents, according to the RECIST (response evaluation criteria in solid tumors) criteria [213], these endpoints may not be optimal in obtaining nuanced information about tumor responses and changes within the tumor microenvironment. For example, in trials with Hsp90-targeted agents, a pertinent endpoint would entail the monitoring of drug-dependent depletion of relevant cancer-causing client proteins. In a recent Phase II trial of 17-AAG in melanoma, differential drug responses were observed for client proteins from patient tumors [214], which may have implications for clinical outcome. Therefore, the ability to dynamically image Hsp90-dependent client depletion *in vivo* would be a clinically valuable addition to these trials. At least two reports demonstrate the feasibility of noninvasively viewing the dynamic expression of Hsp90 client proteins in real time in preclinical models [215, 216], suggesting that incorporation of these approaches into clinical trial design may be feasible.

More recently, a number of clinical trials have included increasingly sophisticated molecular endpoints to gauge drug responses. In a phase II trial of 17-AAG in metastatic prostate cancer, the relation between drug treatment and PSA was monitored, as were a limited number of serum derived cytokines and markers from peripheral blood [131]. In a carefully designed trial of a pan-VEGF inhibitor in GBM, MRI imaging of tumor vascularity discerned a window of vascular normalization [217]. Importantly, this group also identified several vascular modulators that correlated with tumor progression, such as circulating endothelial cells (CECs) and a subset of cytokines, such as SDF-1. In another detailed study by this group, a comprehensive genetic analysis of angiogenic markers revealed that anti-VEGF therapy induced SDF-1 and inflammatory pathways in rectal cancer [218]. This integrative analysis of stage specific molecular and genetic alterations, coupled with the evaluation of surrogate biomarkers, represents a promising approach to better understand and possibly predict fluctuations in the clinical response to Hsp90 inhibitors. Looking forward, it must be determined whether and how Hsp90 inhibitors modulate signaling, angiogenic genes/microRNAs, cytokines, circulating endothelial and progenitor cells, tumor stroma, and which of these alterations are prognostic for tumor response or relapse. Whether the expanded repertoire of proteins targeted by Hsp90 inhibitors, compared with the relatively specific subset of proteins targeted by clinically approved antiangiogenic TKIs, will be an asset or a liability in the clinic remains to be determined. A more comprehensive understanding of the mode of action of Hsp90 inhibitors will position us to harness the power of these agents and design more effective strategies to diminish cancer lethality.
